# Association of Mediterranean diet with survival after breast cancer diagnosis in women from nine European countries: results from the EPIC cohort study

**DOI:** 10.1186/s12916-023-02934-3

**Published:** 2023-06-26

**Authors:** Carlota Castro-Espin, Catalina Bonet, Marta Crous-Bou, Núria Nadal-Zaragoza, Anne Tjønneland, Lene Mellemkjær, Mariem Hajji-Louati, Thérèse Truong, Verena Katzke, Charlotte Le Cornet, Matthias B. Schulze, Franziska Jannasch, Giovanna Masala, Sabina Sieri, Salvatore Panico, Chiara Di Girolamo, Guri Skeie, Kristin Benjaminsen Borch, Karina Standahl Olsen, Maria-Jose Sánchez, Pilar Amiano, María-Dolores Chirlaque, Marcela Guevara, Malin Sund, Stina Bodén, Marc J. Gunter, Esther M. Gonzalez-Gil, Elisabete Weiderpass, Inmaculada Aguilera-Buenosvinos, Kostas K. Tsilidis, Alicia K. Heath, Dagfinn Aune, Laure Dossus, Antonio Agudo

**Affiliations:** 1 Unit of Nutrition and Cancer, Catalan Institute of Oncology-ICO, L’Hospitalet de Llobregat, Barcelona, Spain; 2 Nutrition and Cancer Group, Epidemiology, Public Health, Cancer Prevention and Palliative Care Program, Bellvitge Biomedical Research Institute-IDIBELL, L’Hospitalet de Llobregat, Barcelona, Spain; 3 Department of Epidemiology, Harvard T.H. Chan School of Public Health, Boston, MA 02115 USA; 4 The Danish Cancer Society Research Center, Copenhagen, Denmark; 5 Department of Public Health, The University of Copenhagen, Copenhagen, Denmark; 6 Université Paris-Saclay, UVSQ, Inserm “Exposome, Heredity, Cancer and Health” Team, CESP U1018, Gustave Roussy, Villejuif, France; 7 German Cancer Research Center (DKFZ), Heidelberg, Germany; 8 Department of Molecular Epidemiology, German Institute of Human Nutrition Potsdam-Rehbruecke, Nuthetal, Germany; 9 Institute of Nutritional Science, University of Potsdam, Nuthetal, Germany; 10 Institute for Cancer Research, Prevention and Clinical Network (ISPRO), Florence, Italy; 11 Epidemiology and Prevention Unit, Fondazione IRCCS Istituto Nazionale dei Tumori di Milano Via Venezian, 1. 20133, Milan, Italy; 12 Dipartimento di Medicina Clinica e Chirurgia, Federico II University, Naples, Italy; 13 Centre for Biostatistics, Epidemiology, and Public Health (C-BEPH), Department of Clinical and Biological Sciences, University of Turin, Regione Gonzole 10, Orbassano (TO), Italy; 14 Department of Community Medicine, UiT The Arctic University of Norway, Tromsø, Norway; 15 Escuela Andaluza de Salud Pública (EASP), Granada, 18011 Spain; 16 Instituto de Investigación Biosanitaria ibs.GRANADA, Granada, 18012 Spain; 17 Centro de Investigación Biomédica en Red de Epidemiología y Salud Pública (CIBERESP), Madrid, 28029 Spain; 18 Department of Preventive Medicine and Public Health, University of Granada, Granada, 18071 Spain; 19 Ministry of Health of the Basque Government, Sub Directorate for Public Health and Addictions of Gipuzkoa, San Sebastian, Spain; 20 Biodonostia Health Research Institute, Epidemiology of Chronic and Communicable Diseases Group, San Sebastian, Spain; 21 Department of Epidemiology, Regional Health Council, IMIB-Arrixaca, Murcia University, Murcia, Spain; 22 CIBER in Epidemiology and Public Health (CIBERESP), Madrid, Spain; 23 Instituto de Salud Pública y Laboral de Navarra, Pamplona, 31003 Spain; 24 Navarra Institute for Health Research (IdiSNA), Pamplona, 31008 Spain; 25 Department of Surgical and Perioperative Sciences/Surgery, Umeå University, Umeå, Sweden; 26 Department of Surgery, University of Helsinki & Helsinki University Hospital, Helsinki, Finland; 27 Department of Clinical Sciences/Pediatrics, Umeå University, Umeå, Sweden; 28 International Agency for Research on Cancer, World Health Organization, Lyon, France; 29 Department of Epidemiology and Biostatistics, School of Public Health, Imperial College London, London, UK; 30 Department of Preventive Medicine and Public Health, University of Navarra, Pamplona, Spain; 31 Department of Hygiene and Epidemiology, University of Ioannina School of Medicine, Ioannina, Greece; 32 Department of Nutrition, Oslo New University College, Oslo, Norway; 33 Department of Endocrinology, Morbid Obesity and Preventive Medicine, Oslo University Hospital Ullevål, Oslo, Norway

**Keywords:** Mediterranean diet, Breast cancer, Cancer survivors, Dietary patterns, Prospective studies

## Abstract

**Background:**

The Mediterranean diet has been associated with lower risk of breast cancer (BC) but evidence from prospective studies on the role of Mediterranean diet on BC survival remains sparse and conflicting. We aimed to investigate whether adherence to Mediterranean diet prior to diagnosis is associated with overall and BC-specific mortality.

**Methods:**

A total of 13,270 incident breast cancer cases were identified from an initial sample of 318,686 women in 9 countries from the European Prospective Investigation into Cancer and Nutrition (EPIC) study. Adherence to Mediterranean diet was estimated through the adapted relative Mediterranean diet (arMED), a 16-point score that includes 8 key components of the Mediterranean diet and excludes alcohol. The degree of adherence to arMED was classified as low (score 0–5), medium (score 6–8), and high (score 9–16). Multivariable Cox proportional hazards models were used to analyze the association between the arMED score and overall mortality, and Fine-Gray competing risks models were applied for BC-specific mortality.

**Results:**

After a mean follow-up of 8.6 years from diagnosis, 2340 women died, including 1475 from breast cancer. Among all BC survivors, low compared to medium adherence to arMED score was associated with a 13% higher risk of all-cause mortality (HR 1.13, 95%CI 1.01–1.26). High compared to medium adherence to arMED showed a non-statistically significant association (HR 0.94; 95% CI 0.84–1.05). With no statistically significant departures from linearity, on a continuous scale, a 3-unit increase in the arMED score was associated with an 8% reduced risk of overall mortality (HR_3-unit_ 0.92, 95% CI: 0.87–0.97). This result sustained when restricted to postmenopausal women and was stronger among metastatic BC cases (HR_3-unit_ 0.81, 95% CI: 0.72–0.91).

**Conclusions:**

Consuming a Mediterranean diet before BC diagnosis may improve long-term prognosis, particularly after menopause and in cases of metastatic breast cancer. Well-designed dietary interventions are needed to confirm these findings and define specific dietary recommendations.

**Supplementary Information:**

The online version contains supplementary material available at 10.1186/s12916-023-02934-3.

## Background

Breast cancer (BC) is the most commonly diagnosed cancer and the fifth most common cause of cancer death worldwide, accounting for approximately one in four cancer cases and one in six cancer deaths in 2020 [1, 2]. The survival rate for BC varies depending on the stage of the cancer at the time of diagnosis, age of the patient, and type of BC diagnosed. Despite differences in survival across world regions, with lower rates in transitioning countries than in transitioned countries, early detection and advances in treatment are leading to an increase in the number of BC survivors [3].

Recent reviews show persuasive evidence that body fatness and physical activity may predict important outcomes for patients with breast cancer [4, 5]. However, evidence concerning diet is rather limited or inconclusive, and BC survivors are advised to follow cancer prevention guidelines once their treatment is completed [6, 7].

Adherence to the Mediterranean diet (MD) has been associated with lower risk of breast cancer [8]. Accordingly, most recommendations of the World Cancer Research Fund (WCRF) for cancer survivors are compatible with the MD pattern [4]. However, few studies have explored the role of this dietary pattern in relation to BC survival [9–12]. These studies were conducted in the USA and used slightly different versions of the MD pattern, different dietary assessment methods and collection times, and overall the results were inconclusive.

Our objective was to assess the association between adherence to a MD pattern, by means of the adapted relative Mediterranean diet (arMED) score [8], using dietary data collected prior to diagnosis, and all-cause and BC-specific mortality in women diagnosed with BC from nine European countries.

## Methods

### Study population

We used data from the European Prospective Investigation into Cancer and Nutrition (EPIC) study, a prospective, multicenter European cohort with more than half a million women and men recruited between 1992 and 2000. Full details of the study design and data collection have been described elsewhere [13, 14]. Participants completed questionnaires on diet, lifestyle, and medical history at the time of recruitment and anthropometric measurements were also obtained. All participants provided written informed consent and the study was approved by the ethical review committees of the International Agency for Research on Cancer (IARC-Lyon, France) and all local centers.

### Dietary assessment

The dietary assessment was conducted using a combination of methods, including detailed dietary questionnaires, and food frequency questionnaires (FFQs). At recruitment, participants completed a validated country- or center-specific dietary questionnaire that included questions on the frequency and portion sizes of foods and drinks consumed in the previous year and was designed to capture the geographical specificity of the diet [13].

### Derivation of the arMED score

To measure adherence to the MD, we used the arMED score [8], a variant of the original MD scale defined by Trichopoulou et al. [15]. The arMED score was based on tertiles of energy-adjusted intake of eight foods/food groups to reflect consumption in relation to the individual's total daily energy intake. Unlike the original score [15], the arMED includes olive oil instead of monounsaturated fats, and alcohol was excluded from the list of components due to its causal association with BC carcinogenesis. For five items presumed to fit the MD, a score of 0 to 2 was assigned to tertiles of intake: fruits (including nuts and seeds), vegetables (excluding potatoes), legumes, fish, and cereals. The scoring was inverted for the components presumed to not fit MD: meat (red meat and processed meat) and dairy products. The score was slightly modified for olive oil due to the relatively high proportion of non-consumers in some countries; a score of 0 was assigned to non-consumers, 1 to participants below the median (calculated among consumers), and 2 to participants at or above the median. Thus, the arMED score ranged from 0 to 16, with higher scores indicating greater adherence to MD.

### Ascertainment of breast cancer cases

The International Classification of Diseases for Oncology (ICD-O-2) codes C50.0–50.9 were used to define BC cases. Women with prevalent tumors at recruitment, no follow-up data, no information on lifestyle and diet, or implausible diets were excluded; furthermore, BC cases (*N* = 50) with unknown vital status, inconsistent follow-up data, or with non-epithelial morphology were also excluded. Out of 318,686 women from nine countries (Denmark, France, Germany, Italy, the Netherlands, Norway, Spain, Sweden, and the UK), a total of 13,270 incident primary malignant breast cancers (including 14 in situ) were diagnosed during the follow-up and were included in the present analysis.

### Statistical analyses

The baseline characteristics of the participants were described as mean (SD) for continuous variables and frequencies for categorical variables. Cox proportional hazard models were used to prospectively analyze associations between the arMED score and overall mortality. Fine-Gray competing risks models were performed to evaluate the association with BC-specific mortality, with other causes of death considered competing events. Entry time was defined as the date of diagnosis of primary breast cancer, and exit time was defined as the date of death or end of follow-up. The arMED score was assessed as a categorical variable according to low (score 0–5), medium (score 6–8), and high (score 9–16) adherence, using the medium category as the reference, as well as per 3-unit increase in the score. Restricted cubic spline models with five knots were fitted, and non-linearity was tested using the likelihood (LR) ratio test.

All models were stratified by country, menopausal status at diagnosis (women aged ≥ 55 years at diagnosis were considered postmenopausal regardless of the baseline information) and stage of the tumor (non-metastatic, metastatic, unknown) and adjusted for: age at diagnosis (5-years categories), education level (no formal education, primary school, secondary school, technical or professional training, university, and not specified), body mass index (BMI) (kg/m^2^, continuous), physical activity (inactive, moderately inactive, moderately active, active, unknown), alcohol consumption (non-drinker, 0 to < 3 g/day, 3 to < 12 g/day, 12 to < 24 g/day, ≥ 24 g/day, unknown), smoking status and intensity (never smokers, current smokers 1–15, 16–25, and > 25 cigarettes/day, former quit ≤ 10, 11–20, and > 20 years before recruitment, current smoker of cigars, pipes and occasional current smokers, current smokers with unknown intensity, and not specified), ever use of hormone replacement therapy for menopause at diagnosis (yes, no, unknown), grade of tumor (well differentiated, moderately differentiated, poorly differentiated or undifferentiated, not determined), and tumor receptor status (positive, negative, unknown) for estrogen receptor (ER), progesterone receptor (PR), and human epidermal growth factor receptor 2 (HER2). BMI was modeled as a restricted cubic spline to account for its non-linear association with mortality [5, 16].

Separate models for pre- and postmenopausal cases were performed and heterogeneity was tested by comparing models with and without the cross-product terms using the Likelihood ratio (LR) test. The proportional hazards assumptions were tested by using the Schoenfeld goodness-of-fit test.

Stratified analyses were performed for overall mortality according to potential modifiers of the association with arMED: BMI, physical activity, smoking status, tumor stage, and hormone receptor status (ER, PR, HER2, and triple negative), and adherence to dietary patterns related to underlying biological mechanisms of BC previously associated with BC survival [17]: low/high adherence to the Diabetes Risk Reduction Diet [DRRD] [18] and Inflammatory Score of Diet [ISD] [19].

In sensitivity analyses, we examined whether further adjustment for the time interval between recruitment (time at which dietary information was collected) and BC diagnosis modified our main results, as well as for the period of diagnosis, to account for the potential influence of improvements in treatment and diagnosis over time. Comorbidities, including cardiovascular disease and presence of diabetes at baseline, and a combined variable with mechanistic dietary patterns (DRRD-ISD) were also used to further adjust separate models and test the robustness of the results.

Direct adjusted cumulative incidence function (CIF) curves for the three levels of adherence to the arMED score and overall mortality were derived from the multivariable Cox proportional hazards model [20].

All analyses were performed using R version 4.2.2. We used a significance level of 0.05, but also considered the confidence intervals and point estimate magnitudes. Data analysis was conducted from October 1, 2022, to January 13, 2023.

## Results

Our population included 13,270 incident cases of breast cancer. During a mean follow-up of 8.6 years from the date of diagnosis, 2340 women died, including 1475 specifically from breast cancer. Baseline characteristics of the women in relation to three levels of adherence to the arMED score are summarized in Table 1. Women with low arMED were older, with lower educational level, more likely to be current smokers, more likely to have a higher BMI, and were mostly postmenopausal. Women with low adherence to the arMED were also less likely to have well-differentiated tumors, more likely to be diagnosed at an earlier stage, and with a higher proportion of ER-positive and PR-positive tumors. Mediterranean countries in the cohort had the highest arMED scores (Spain, Italy, mean 10), and lowest for Sweden and the Netherlands (mean 5) (Additional file 1: Table S1). The adjusted HRs for overall and BC-specific mortality according to levels of adherence to the arMED score are summarized in Table 2. Among all BC survivors, low compared to medium adherence to arMED was associated with a 13% higher risk of all-cause mortality (HR 1.13, 95% CI: 1.01–1.26). Compared to medium adherence, the association for high adherence was not statistically significant (HR 0.94; 95% CI 0.84–1.05). Despite this, an assessment of the association of arMED and overall and BC-specific mortality using restricted cubic splines (Additional file 1: Fig. S1) showed no statistically significant departures from linearity (*p* = 0.8). Therefore, on a continuous scale, a 3-unit increase in the arMED score was associated with an 8% (HR_3-unit_ 0.92, 95% CI: 0.87–0.97) reduced risk of overall mortality. In postmenopausal BC survivors, the arMED score showed an 8% (HR_3-unit_ 0.92, 95% CI: 0.86–0.98) lower risk of overall mortality per 3-unit increase, whereas there was no statistically significant association in premenopausal BC survivors (HR_3-unit_ 0.94, 95% CI: 0.84–1.06). Heterogeneity between the association of arMED and overall mortality according to menopausal status did not reach statistical significance (*p* = 0.437). Multivariable models for BC-specific mortality showed no evidence of association with the arMED score, in all BC survivors and in neither premenopausal nor postmenopausal BC survivors. Stratified analyses by Mediterranean and non-Mediterranean countries (Additional file 1: Table S2) showed overall inverse associations by increasing the arMED score and overall mortality with stronger associations among Mediterranean (HR_3-unit_ 0.81, 95% 0.69–0.95) countries and no longer statistically significant among non-Mediterranean countries.

**Table 1:** Baseline characteristics of the 13,270 breast cancer (BC) cases in the EPIC cohort according to the adherence to Mediterranean diet as measured by the adapted relative Mediterranean diet (arMED) score

	All BC cases (*N* = 13,270)	Adherence measured by the arMED score		
		Low (*N* = 3427)	Medium (*N* = 5162)	High (*N* = 4681)
Age at diagnosis, mean (SD)	61 (8.8)	63 (8.8)	61 (8.5)	60 (8.9)
Educational level				
None/primary	3281 (24.7)	998 (29.1)	1049 (20.3)	1234 (26.4)
Technical/professional	3035 (22.9)	1148 (33.5)	1242 (24.1)	645 (13.8)
Secondary	3200 (24.1)	622 (18.1)	1317 (25.5)	1261 (26.9)
Longer education	3143 (23.7)	588 (17.2)	1294 (25.1)	1261 (26.9)
Smoking status				
Never	5940 (44.8)	1465 (42.7)	2276 (44.1)	2199 (47)
Former	3139 (23.7)	806 (23.5)	1233 (23.9)	1100 (23.5)
Current	2476 (18.7)	895 (26.1)	936 (18.1)	645 (13.8)
Miscellaneous	1487 (11.2)	236 (6.9)	605 (11.7)	646 (13.8)
Alcohol consumption				
Non drinker	1774 (13.4)	422 (12.3)	598 (11.6)	754 (16.1)
> 0–3 g/day	3820 (28.8)	990 (28.9)	1455 (28.2)	1375 (29.4)
> 3–12 g/day	4066 (30.6)	1035 (30.2)	1641 (31.8)	1390 (29.7)
> 12–24 g/day	2069 (15.6)	523 (15.3)	801 (15.5)	745 (15.9)
> 24 g/day	1541 (11.6)	457 (13.3)	667 (12.9)	417 (8.9)
Body mass index (BMI)				
Normal weight	7612 (57.4)	1834 (53.5)	3058 (59.2)	2720 (58.1)
Overweight	3943 (29.7)	1105 (32.2)	1473 (28.5)	1365 (29.2)
Obesity	1509 (11.4)	440 (12.8)	556 (10.8)	513 (11)
Underweight	206 (1.6)	48 (1.4)	75 (1.5)	83 (1.8)
Physical activity				
Inactive	2673 (20.1)	583 (17)	891 (17.3)	1199 (25.6)
Moderately inactive	4720 (35.6)	1200 (35)	1820 (35.3)	1700 (36.3)
Moderately active	3606 (27.2)	867 (25.3)	1555 (30.1)	1184 (25.3)
Active	2076 (15.6)	709 (20.7)	809 (15.7)	558 (11.9)
Menopausal status at diagnosis				
Premenopausal	3070 (23.1)	613 (17.9)	1152 (22.3)	1305 (27.9)
Postmenopausal	10,200 (76.9)	2814 (82.1)	4010 (77.7)	3376 (72.1)
Ever use of hormones for menopause				
No	7487 (56.4)	1785 (52.1)	2805 (54.3)	2897 (61.9)
Yes	5323 (40.1)	1434 (41.8)	2166 (42)	1723 (36.8)
Unknown	460 (3.5)	208 (6.1)	191 (3.7)	61 (1.3)
Grade of tumor differentiation				
Well differentiated	1298 (9.8)	269 (7.8)	547 (10.6)	482 (10.3)
Moderately differentiated	2917 (22)	603 (17.6)	1176 (22.8)	1138 (24.3)
Undifferentiated or poorly diff	2503 (18.9)	462 (13.5)	982 (19)	1059 (22.6)
Not determined	6552 (49.4)	2093 (61.1)	2457 (47.6)	2002 (42.8)
Stage of tumor				
Stage 0/I	1954 (14.7)	573 (16.7)	798 (15.5)	583 (12.5)
Stage II	1593 (12)	506 (14.8)	624 (12.1)	463 (9.9)
Stage III	303 (2.3)	98 (2.9)	119 (2.3)	86 (1.8)
Non-metastatic, unknown stage	3984 (30)	773 (22.6)	1622 (31.4)	1589 (33.9)
Stage IV (metastatic)	1777 (13.4)	350 (10.2)	699 (13.5)	728 (15.6)
Unknown	3659 (27.6)	1127 (32.9)	1300 (25.2)	1232 (26.3)
ER status				
Negative	1678 (12.6)	391 (11.4)	646 (12.5)	641 (13.7)
Positive	7500 (56.5)	1739 (50.7)	2968 (57.5)	2793 (59.7)
Unknown	4092 (30.8)	1297 (37.8)	1548 (30)	1247 (26.6)
PR status				
Negative	2612 (19.7)	571 (16.7)	1029 (19.9)	1012 (21.6)
Positive	5072 (38.2)	1129 (32.9)	1983 (38.4)	1960 (41.9)
Unknown	5586 (42.1)	1727 (50.4)	2150 (41.7)	1709 (36.5)
HER2 status				
Negative	3587 (27)	931 (27.2)	1377 (26.7)	1279 (27.3)
Positive	856 (6.5)	202 (5.9)	312 (6)	342 (7.3)
Unknown	8827 (66.5)	2294 (66.9)	3473 (67.3)	3060 (65.4)

Except for values where the mean and SD are specified, all values are presented as the total number (*N*) and %

Cut-off points of arMED categories: low adherence, 0–5; medium adherence, 6–8; high adherence, 9–16

Unknown categories of educational level (*N* = 611), smoking habit and intensity (*N* = 228), and physical activity (*N* = 195) are not shown in this table

**Table 2:** Multivariable hazard ratios (HR) and 95% confidence interval (95%CI) of overall and breast cancer-specific mortality according to the adherence to the Mediterranean diet measured by the arMED score

	Categories of adherence of the arMED score^a^			arMED score, continuous
	Low	Medium	High	3-units increase
**Overall mortality**				
All BC survivors^b^	1.13 (1.01–1.26)	1.00 reference	0.94 (0.84–1.05)	0.92 (0.87–0.97)
* N* (deaths)	3427 (736)	5162 (871)	4681 (733)	13,270 (2340)
Premenopausal BC survivors^c,d^	1.21 (0.94–1.56)	1.00 reference	1.11 (0.89–1.38)	0.94 (0.84–1.06)
*N* (deaths)	613 (131)	1152 (183)	1305 (213)	3070 (527)
Postmenopausal BC survivors^c,e^	1.10 (0.98–1.24)	1.00 reference	0.89 (0.78–1.01)	0.92 (0.86–0.98)
*N* (deaths)	2814 (605)	4010 (688)	3376 (520)	10,200 (1813)
*P* value heterogeneity^*^				0.437
**BC-specific mortality**				
All BC survivors^b^	1.12 (0.98–1.29)	1.00 reference	0.99 (0.86–1.13)	0.97 (0.90–1.04)
*N* (deaths)	3427 (441)	5162 (541)	4681 (493)	13,270 (1475)
Premenopausal BC survivors^c,d^	1.23 (0.92–1.63)	1.00 reference	1.16 (0.91–1.48)	0.97 (0.85–1.11)
*N* (deaths)	613 (103)	1152 (145)	1305 (180)	3070 (428)
Postmenopausal BC survivors^e^	1.10 (0.94–1.28)	1.00 reference	0.93 (0.78–1.09)	0.97 (0.89–1.05)
*N* (deaths)	2814 (338)	4010 (396)	3376 (313)	10,200 (1047)
*P* value heterogeneity^*^				0.992

Abbreviations: *arMED* Adapted relative Mediterranean diet, *N* Number of breast cancer cases

^a^Categories of arMED: low adherence, 0–5; medium adherence, 6–8; high adherence, 9–16

^b^Model stratified by country, stage (metastatic, non-metastatic, unknown) and menopausal status at diagnosis and adjusted for age at diagnosis, attained level of education, physical activity, body mass index (modeled as restricted cubic spline), alcohol consumption reported at recruitment, smoking habit and intensity at recruitment, ever use of hormones for menopause at diagnosis, grade of differentiation, and tumor receptor status (ER, PR, HER2)

^c^Same model as above without stratification for menopausal status al diagnosis

^d^Model premenopause: variable age into 2 categories, < 50 and >  = 50, and not adjusted for every use of hormone therapy replacement for menopause

^e^Model postmenopause: variable age into 2 categories, < 65 and >  = 65

^*^Pvalue for heterogeneity between pre- and postmenopausal subgroups for the association of arMED and mortality outcomes

**Fig. 1:**
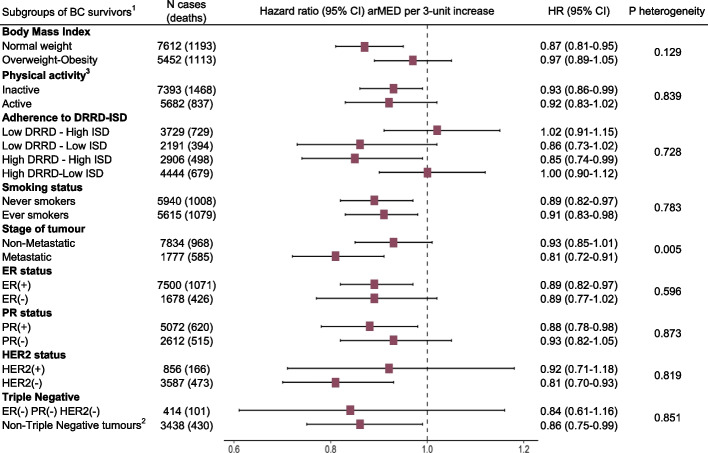
Associations between arMED score and overall survival among subgroups of breast cancer survivors. Abbreviations: BC, breast cancer; BMI, body mass index; HR, hazard ratio; CI, confidence interval; ER, estrogen receptor; PR, progesterone receptor; HER2, human epidermal growth factor receptor 2; DRRD, Diabetes Risk Reduction Diet; ISD, Inflammatory Score of the Diet; *N*, number of breast cancer cases. ^1^Each model has excluded of their stratification/adjustment the specific variable that represents the subgroup in each case. Models stratified by country, stage (metastatic, non-metastatic, unknown) and menopausal status at diagnosis and adjusted for age at diagnosis, attained level of education, physical activity, body mass index (modeled as restricted cubic spline with five internal knots placed at equally spaced percentiles), alcohol consumption reported at recruitment, smoking habit and intensity as cigarettes per day at recruitment, ever use of hormones for menopause at diagnosis, grade of differentiation, and tumor receptor status: ER, PR, HER2. ^2^Only BC survivors with known status of the receptor (positive or negative) have been considered. ^3^Categories of physical activity. Inactive: includes moderately inactive and inactive. Active: includes moderately active and active

Results for arMED and overall mortality were unchanged with further adjustment for time between dietary data collection and BC diagnosis, period of diagnosis, or presence of comorbidities at baseline (Table 3). However, adjustment for adherence to DRRD-ISD dietary patterns attenuated the association. We also examined the association between arMED and non-BC-related deaths, including deaths from CVD and cancers other than BC (Additional file 1: Table S3). The arMED score was not associated with CVD mortality, whereas a statistically significant inverse association was observed for total non-BC-related mortality (*p* < 0.01) and cancer mortality (excluding BC) (*p* < 0.01). Figure 1 shows subgroup analyses for arMED per 3-unit increase in relation to overall mortality. Although there were clear associations in some subgroups but not others, there were no statistically significant interactions for the association between arMED score and overall mortality with BMI, physical activity, adherence to the DRRD-ISD dietary patterns, smoking status, or hormone receptor status, including triple negative tumors.

**Table 3:** Sensitivity analysis: association between overall mortality and adherence to the arMED score by additional adjustments for time from baseline to diagnosis, period of diagnosis, presence of comorbidities, and other dietary patterns (*N* = 13,270, events/deaths = 2340)

	Categories of adherence of the arMED score^a^			arMED score, continuous
	Low (*N* = 3427)	Medium (*N* = 5162)	High (*N* = 4681)	3-units increase
Reference model^b^	1.13 (1.01–1.25)	1.00 Reference	0.94 (0.84–1.05)	0.92 (0.87–0.97)
Model further adjusted for:				
Time from baseline to diagnosis^c^	1.13 (1.02–1.26)	1.00 Reference	0.94 (0.84–1.04)	0.92 (0.87–0.97)
Period of diagnosis^d^	1.13 (1.01–1.25)	1.00 Reference	0.94 (0.84–1.05)	0.92 (0.87–0.97)
Comorbidities^e^	1.13 (1.02–1.26)	1.00 Reference	0.93 (0.84–1.04)	0.92 (0.86–0.97)
Mechanistic dietary patterns (DRRD-ISD)^f^	1.10 (0.99–1.23)	1.00 Reference	0.98 (0.87–1.09)	0.95 (0.89–1.01)

Abbreviations: *arMED* Adapted relative Mediterranean diet, *N* Number of breast cancer cases

^a^Categories of arMED: low adherence, 0–5; medium adherence, 6–8; high adherence, 9–16

^b^Model stratified by country, stage (metastatic, non-metastatic, unknown) and menopausal status at diagnosis and adjusted for age at diagnosis, attained level of education, physical activity, body mass index (modeled as restricted cubic spline), alcohol consumption reported at recruitment, smoking habit and intensity at recruitment, ever use of hormones for menopause at diagnosis, grade of differentiation, and tumor receptor status (ER, PR, HER2)

^c^Categories of time from baseline to diagnosis are < 5 years, 5 to < 8 years, 8 to < 12 years, and equal or more than 12 years

^d^Categories for period of diagnosis are before 2000, between 2000 and < 2004, between 2004 and < 2008, 2008 onwards

^e^Comorbidities include the presence of diabetes (categories yes, no, unknown) and the presence of cardiovascular problem reported at recruitment (categories yes, no, unknown)

^f^Adjusted for a 4-level variable that combines two dietary patterns in high-low adherence categories: Diabetes Reduced Risk Diet (DRRD) and Inflammatory Score of Diet (ISD)

**Fig. 2:**
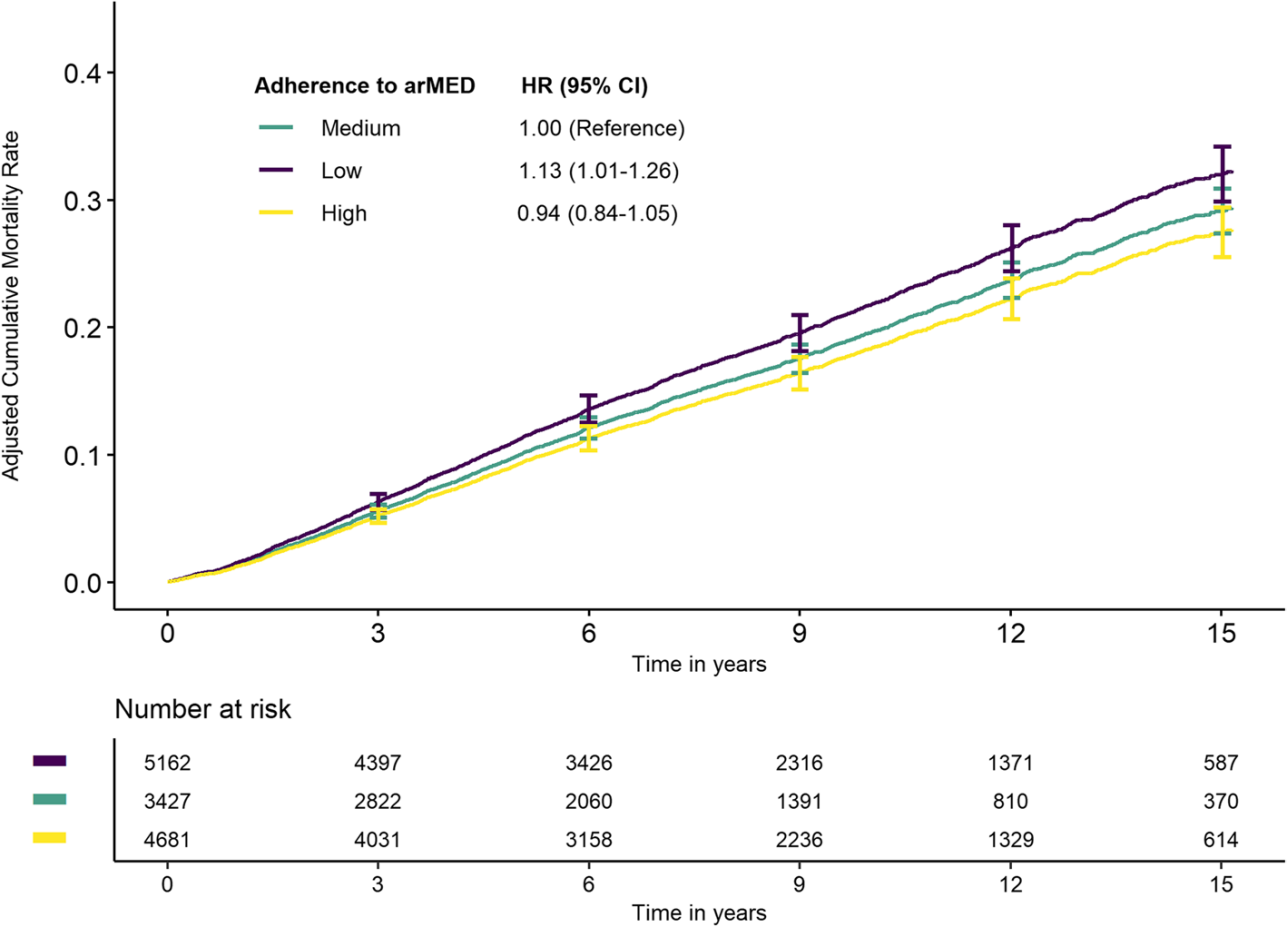
Direct adjusted cumulative incidence function curves for overall mortality by categories of adherence to arMED score. HR hazard ratio, CI confidence interval, arMED adapted relative Mediterranean diet. Categories of arMED: low adherence, 0–5; medium adherence, 6–8; high adherence, 9–16. HRs and CI 95% from multivariable Cox model stratified by country, stage (metastatic, non-metastatic, unknown) and menopausal status at diagnosis and adjusted for age at diagnosis, attained level of education, physical activity, body mass index (modeled as restricted cubic spline), alcohol consumption reported at recruitment, smoking habit and intensity at recruitment, ever use of hormones for menopause at diagnosis, grade of differentiation, and tumor receptor status (ER, PR, HER2)

The only statistically significant interaction was observed with tumor stage at diagnosis (non-metastatic versus metastatic tumors) (*p* < 0.01): a protective association of arMED was observed in metastatic BC survivors for overall mortality (HR_3-unit_ 0.81, 95% CI: 0.72–0.91): The same was observed for BC-specific mortality, with HR_3-unit_ 0.86 (95% CI: 0.76–0.98), with *p*-value heterogeneity < 0.01 (Additional file 1: Table S4). The direct adjusted CIF curves for overall mortality derived from the Cox model are presented in Fig. 2. The 15-year cumulative mortality estimates were 32% (30–34%), 29% (27–31%), and 27% (26–29%) for low, medium, and high adherence to the arMED score, respectively.

## Discussion

In this prospective cohort study including 13,270 BC survivors, low adherence to a MD pattern before BC diagnosis was positively associated with all-cause mortality (13% higher risk compared to medium adherence). The results also showed an 8% lower overall mortality in all BC survivors for each 3-unit increase in the arMED score, which was sustained when restricted to postmenopausal women. Although no significant heterogeneity was observed by menopausal status, the smaller number of premenopausal cases may have limited our statistical power to detect significant associations. Additionally, while a statistically significant lower risk of all-cause mortality was observed with the arMED score modeled continuously, the association did not reach statistical significance when comparing high versus medium adherence. Moreover, there were no clear associations for BC-specific mortality. In addition, high adherence to MD appears to be particularly beneficial against overall mortality in Mediterranean countries, whereas in non-Mediterranean countries, the impact of increased adherence to MD seems more relevant when moving from low to medium levels of adherence.

Two recent systematic reviews have evaluated the relationship of several dietary patterns with BC survival [21, 22]. Only three studies included in these reviews evaluated the role of a MD pattern on prognostic outcomes in BC survivors [9–11]. Our study found a statistically significant inverse association between adherence to MD and all-cause mortality which conflicts with the null results in these studies. This discrepancy could be owing to a number of differences, including the study population, the score used to measure adherence to MD, the time when diet was measured with respect to BC diagnosis, and the different method of dietary assessment. For instance, the three studies [9–11], conducted in the USA, did not consider olive oil, but used a ratio of monounsaturated to saturated fats instead, and all of them included alcohol consumption. One study was restricted to postmenopausal women with stage I-III BC [9], and another [10] was a retrospective cohort with a small sample size (*N* = 110) of self-reported BC survivors whose dietary information was based on a single 24-h dietary recall. Finally, whereas our study assessed diet before diagnosis, other studies did it either close to the time of diagnosis [11] or after diagnosis [9].

Despite this, and in line with our results, a recent study observed statistically significant inverse associations with overall mortality for higher adherence to the Alternative Mediterranean Diet (aMED), a version of the MD adapted to the US population [12]. However, the Breast Cancer Family Registry is based on families with multiple cases of BC and the findings may not be generalizable to women without a family history of BC.

In our study, 63% of deaths were related specifically to BC, and BC used to be the most common cause of death following a BC diagnosis [23]. However, evidence now suggests that cardiovascular disease (CVD) accounts for a substantial number of non-BC-related deaths in the 10 years following diagnosis [23, 24]. Although MD is well-known to be protective against CVD [25], in our study, most deaths by causes other than BC were related to other cancers (13%) and only 150 (6%) were from CVD (Additional file 1: Table S3). Our findings showed statistically significant inverse associations between arMED score and overall non-BC mortality, including cancer mortality (excluding BC), but a non-significant association with CVD-related mortality. This warrants further investigation in future studies with a greater number of deaths related to these causes. On the other hand, Cox models additionally adjusted for the presence of comorbidities, including CVD or diabetes at the time of recruitment, showed no apparent differences with respect to the main results (Table 2), suggesting that the effect of the MD on mortality could occur beyond its protective role on these diseases.

Consistent with our results, the Nurses’ Health Study found a statistically significant reduced risk of all-cause mortality with higher adherence to MD restricted to BC survivors with low physical activity [9]. Our subgroup analyses also showed a statistically significant inverse association between adherence to MD and all-cause mortality in BC survivors who were inactive, and additionally in those with normal weight. Similarly, a recent study reported a statistically significant inverse association for adherence to MD with overall mortality in BC cases of normal BMI range [12]. A plausible interpretation for this might be that the impact of diet is more evident in BC survivors without the presence of other strong prognostic determinants such as obesity and physical activity, where perhaps the mechanisms underlying these conditions may blur the modest effect of diet. However, no heterogeneity was found, hence no firm conclusions can be drawn.

With respect to hormone receptors, BC survivors with ER/PR-positive and HER2-negative tumors showed lower overall mortality with increasing arMED score, although there was no statistically significant interaction between groups (*p* > 0.05). Metastatic BC survivors seemed to benefit more from adherence to the MD when assessed by the arMED score than non-metastatic BC survivors, with a statistically significant reduced risk of overall mortality and BC-specific mortality, however, we do not have a clear interpretation for these results.

The positive effect on health attributed to the MD [26] is thought to be due to several mechanisms, including weight control, antioxidant potential, improvement of glycemic profile, and anti-inflammatory properties [27, 28]. Some of these mechanisms are also involved in the prognosis of breast cancer [29]. However, to our knowledge, the impact of the MD pattern on these mechanisms in relation to BC prognosis has rarely been investigated [30]. We have recently reported that two dietary patterns related to lower insulin resistance and the inflammatory potential of the diet (the DRRD and the ISD, respectively) were associated with overall mortality among BC survivors [17]. To investigate the independent effect of the arMED score we adjusted a model with a variable combining high and low adherence to the DRRD and ISD scores. We found that the observed association of the arMED with mortality was attenuated, probably reflecting that, at least partially, the observed effect of MD may be explained by mechanisms of inflammation and insulin resistance or hyperinsulinemia.

## Strengths and limitations

This study has several strengths. The prospective study design and long follow-up time allowed us to identify large numbers of deaths among a large sample of BC survivors. One specific advantage of this study is the inclusion of populations from Mediterranean and non-Mediterranean countries, which allowed us to have a broad representation of degrees of adherence to the MD pattern, with enough participants (and events) in different categories of the arMED.

One important limitation is the lack of information on treatment, a strong determinant of prognosis and survival. We used the available information on tumor stage at diagnosis, grade of tumor differentiation, and hormone receptor status as potential surrogates for treatment, since these characteristics are strong determinants of the therapeutic strategy, at least in most settings where the EPIC cohorts were recruited. Although we investigated differences by hormone receptor status for BC mortality outcomes, estimates were imprecise probably due to the missingness among these variables in our analysis. In addition, our results are based on a single dietary assessment, which may not reflect possible changes in dietary habits. To address to what extent the diet collected at baseline remains constant until diagnosis we performed sensitivity analyses adjusting for the time from diet measurement to diagnosis, and the results were unchanged. Similarly, we considered the possibility that survivors with an older diagnosis would be less likely to benefit from the early detection and advances in treatments, resulting in a worse prognosis, as compared to those with a more recent diagnosis. We performed sensitivity analyses adjusting for different periods of diagnosis and the observed results remained stable.

## Conclusions

Our research suggests that adherence to a Mediterranean diet before BC diagnosis may improve long-term prognosis, especially in postmenopausal women. Furthermore, we found that the protective effect of MD appears to be stronger in women diagnosed with metastatic BC tumors. The link between postdiagnosis dietary patterns and BC outcomes remains unclear and BC survivors are still advised to follow general guidelines for cancer prevention once their treatment is completed. Further research, including large, well-designed dietary interventions will help provide more specific dietary recommendations for breast cancer survivors. In the meantime, research on the underlying biological mechanisms of various dietary patterns may provide relevant insights on the role of nutrition in breast cancer prognosis.

## Supplementary Information

**Additional file 1.** Association of Mediterranean diet with survival after breast cancer diagnosis in women from nine European countries: results from the EPIC cohort study. **Table S1.** Summary of the arMED score in women from different EPIC countries. **Table S2.** Multivariable hazard ratiosand 95% confidence intervalof risk of overall mortality according to adherence to the Mediterranean diet across Mediterranean and non-Mediterranean countries in the EPIC study. **Table S3.** Multivariable hazard ratiosand 95% confidence intervalfor the adherence to the Mediterranean diet measured by the arMED score and other causes of death in all BC survivors. **Table S4.** Associations between the arMED score and BC-specific survival among breast cancer cases with non-metastatic and metastatic tumours. **Figure S1.** Assessment of linear associations between arMED score and overall and BC-specific mortality using restricted cubic spline models.

## Data Availability

EPIC data are available for investigators who seek to answer important questions on health and disease in the context of research projects that are consistent with the legal and ethical standard practices of IARC/WHO and the EPIC centers. The primary responsibility for accessing the data belongs to IARC and the EPIC centers. For information on how to submit an application for gaining access to EPIC data and/or biospecimens, please follow the instructions at https://login.research4life.org/tacsgr0epic_iarc_fr/access/index.php.

## Abbreviations

arMED: Adapted relative Mediterranean diet
BC: Breast cancer
BMI: Body mass index
CI: Confidence interval
DRRD: Diabetes Risk Reduction Diet
EPIC: European Prospective Investigation into Cancer and Nutrition
ER: Estrogen receptor
HER2: Human epidermal growth factor receptor 2
HR: Hazard ratio
ISD: Inflammatory score of the diet
MD: Mediterranean diet
PR: Progesterone receptor
SD: Standard deviation
