# Supplementary material for: Association of Mediterranean diet with survival after breast cancer diagnosis in women from nine European countries: results from the EPIC cohort study
Source: BMC Med. 2023 Jun 26;21:225. doi: 10.1186/s12916-023-02934-3 (PMC10294413; doi:10.1186/s12916-023-02934-3)
Supplement: Supplementary file 1 — Additional file 1. Association of Mediterranean diet with survival after breast cancer diagnosis in women from nine European countries: results from the EPIC cohort study. Table S1. Summary of the arMED score in women from different EPIC countries. Table S2. Multivariable hazard ratiosand 95% confidence intervalof risk of overall mortality according to adherence to the Mediterranean diet across Mediterranean and non-Mediterranean countries in the EPIC study. Table S3. Multivariable hazard ratiosand 95% confidence intervalfor the adherence to the Mediterranean diet measured by the arMED score and other causes of death in all BC survivors. Table S4. Associations between the arMED score and BC-specific survival among breast cancer cases with non-metastatic and metastatic tumours. Figure S1. Assessment of linear associations between arMED score and overall and BC-specific mortality using restricted cubic spline models. [file 12916_2023_2934_MOESM1_ESM.docx]

Additional file 1

Association of Mediterranean diet with survival after breast cancer diagnosis in women from nine European countries: results from the EPIC cohort study:

Supplementary Tables S1-S4 and Figure S1.

| **Table S1**. Summary of the arMED score in women from different EPIC countries (N=318686). | | | |  |  |
| --- | --- | --- | --- | --- | --- |
|  |  |  |  |  |  |
| **Country** | **BC survivors** | **Mean (SD)** | **Median (P25,P75)** | **Range** |  |
| France | 3317 | 8.2 (2.2) | 8 (7,10) | (0 - 16) |  |
| Italy | 1200 | 10.2 (2.2) | 10 (9,12) | (1 - 16) |  |
| Spain | 653 | 10.1 (2.6) | 10 (9,12) | (1 - 16) |  |
| United Kingdom | 1875 | 8.6 (2.4) | 9 (7,10) | (0 - 15) |  |
| The Netherlands | 1034 | 5.1 (1.9) | 5 (4,6) | (0 - 14) |  |
| Germany | 814 | 6.0 (2.1) | 6 (5,7) | (0 - 15) |  |
| Sweden | 1312 | 5.2 (2.1) | 5 (4,7) | (0 - 14) |  |
| Denmark | 1865 | 5.9 (2.3) | 6 (4,7) | (0 - 16) |  |
| Norway | 1200 | 7.5 (2.0) | 7 (6,9) | (0 - 13) |  |
| Total | 13270 | 7.6 (2.2) | 8 (6,10) | (0 - 16) |  |

Abbreviations: N number, BC breast cancer, P percentile

| **Table S2.** Multivariable hazard ratios (HR) and 95% confidence interval (95%CI) of risk of overall mortality according to adherence to the Mediterranean diet across Mediterranean and non-Mediterranean countries in the EPIC study. | | | | |
| --- | --- | --- | --- | --- |
|  |  |  |  |  |
| Overall mortality | Categories of adherence of the arMED score^1^ HR (95%CI) | | | arMED score HR (95%CI) |
| Countries | Low | Medium | High | 3-units increase |
| **Mediterranean (arMED score >9)**^4^ | 1.00 (0.48-2.09) | 1.00 (Ref) | 0.74 (0.55-0.99) | 0.81 (0.69-0.95) |
| N (deaths) | 41 (9) | 379 (64) | 1433 (214) | 1853 (287) |
| **Non-Mediterranean (arMED score 7-9)**^4^ | 1.14 (0.95-1.37) | 1.00 (Ref) | 0.96 (0.84-1.1) | 0.94 (0.87-1.03) |
| N (deaths) | 810 (166) | 2820 (449) | 2762 (425) | 6392 (1040) |
| **Non-Mediterranean (arMED score <7)**^4^ | 1.15 (1.00-1.31) | 1.00 (Ref) | 1.06 (0.84-1.34) | 0.94 (0.86-1.03) |
| N (deaths) | 2576 (561) | 1963 (358) | 486 (94) | 5025 (1013) |
| ^1^Categories of arMED: Low adherence, 0-5; Medium adherence, 6-8; High adherence, 9-16. | | | | |
| ^2^Model stratified by stage (metastatic, non-metastatic, unknown) and menopausal status at diagnosis and adjusted for age at diagnosis, attained level of education, physical activity, body mass index (modelled as restricted cubic spline), alcohol consumption reported at recruitment, smoking habit and intensity at recruitment, ever use of hormones for menopause at diagnosis, grade of differentiation, and tumour receptor status (ER, PR, HER2). | | | | |
| ^3^Non-Mediterranean countries are all except Spain and Italy. | |  |  |  |
| ^4^Mediterranean countries (arMED score >9) = Spain and Italy; Non-Mediterranean countries (arMED 7-9) = France, United Kingdom, Norway; Non-Mediterranean countries (arMED <7) = The Netherlands, Germany, Sweden, Denmark. | | | | |

| **Table S3**. Multivariable hazard ratios (HR) and 95% confidence interval (95%CI) for the adherence to the Mediterranean diet measured by the arMED score and other causes of death in all BC survivors. | | | | |
| --- | --- | --- | --- | --- |
|  |  |  |  |  |
|  | Categories of adherence of the arMED score^1^ | | | arMED score, continuous |
|  | Low (N=3427) | Medium (N=5162) | High (N=4681) | 3-units increase (N=13270) |
| **Non-BC-related death** | 1.14 (0.96-1.36) | 1.00 Reference | 0.87 (0.73-1.05) | 0.85 (0.77-0.93) |
| N deaths | 295 | 330 | 240 | 865 |
| **Cancer-related deaths (excluding BC)** | 1.13 (0.85-1.49) | 1.00 Reference | 0.77 (0.56-1.04) | 0.82 (0.70-0.95) |
| N deaths | 110 | 122 | 83 | 315 |
| **CVD-related deaths** | 0.83 (0.55-1.26) | 1.00 Reference | 0.88 (0.56-1.38) | 0.91 (0.72-1.15) |
| N deaths | 46 | 65 | 39 | 150 |
| CVD cardiovascular diseases; arMED alternative relative Mediterranean diet; HR Hazard Ratio; CI confidence interval, BC breast cancer, N number. | | | | |
| ^1^Categories of arMED: Low adherence, 0-5; Medium adherence, 6-8; High adherence, 9-16. | | | | |
| ^2^Model stratified by country, stage (metastatic, non-metastatic, unknown) and menopausal status at diagnosis and adjusted for age at diagnosis, attained level of education, physical activity, body mass index (modelled as restricted cubic spline), alcohol consumption reported at recruitment, smoking habit and intensity at recruitment, ever use of hormone for menopause at diagnosis, grade of differentiation, and tumour receptor status (ER, PR, HER2). | | | | |
| ^3^The three top cancers were from lung (code C34, n=55), colorectal (codes C18-20, n=40), and pancreas (code C25, n=29). | | | |  |

| **Table S4.** Associations between the arMED score and BC-specific survival among breast cancer cases with non-metastatic and metastatic tumours. | | | | | |
| --- | --- | --- | --- | --- | --- |
|  |  |  |  |  |  |
| BC-Specific Mortality | | N cases | Deaths | Hazard ratio (95% CI) arMED per 3-unit increase | P Heterogeneity |
| Stage of tumour | |  |  |  |  |
|  | Non-Metastatic | 7834 | 564 | 0.95 (0.85-1.07) | 0.009 |
|  | Metastatic | 1777 | 449 | 0.86 (0.76-0.98) |  |
| Abbreviations: arMED, adapted relative Mediterranean diet; N, number of breast cancer cases; CI, confidence interval. | | | | | |
| Categories of arMED: Low adherence, 0-5; Medium adherence, 6-8; High adherence, 9-16. | | | | |  |
| ^1^Model stratified by country and menopausal status at diagnosis and adjusted for age at diagnosis, attained level of education, physical activity, body mass index (modelled as restricted cubic spline), alcohol consumption reported at recruitment, smoking habit and intensity at recruitment, ever use of hormones for menopause at diagnosis, grade of differentiation, and tumour receptor status (ER, PR, HER2). | | | | | |


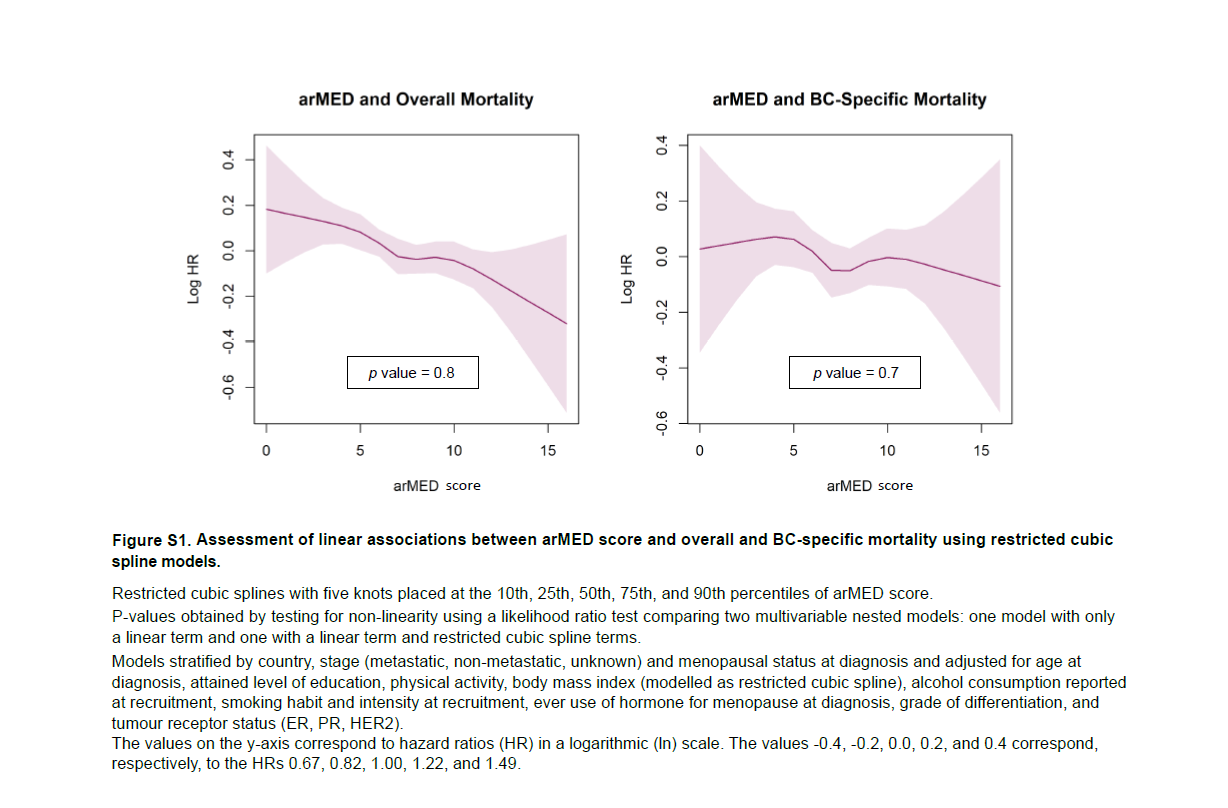


**Figure S1**. Assessment of linear associations between arMED score and overall and BC-specific mortality using restricted cubic spline models.

Restricted cubic splines with five knots placed at the 10th, 25th, 50th, 75th, and 90th percentiles of arMED score.

P-values obtained by testing for non-linearity using a likelihood ratio test comparing two multivariable nested models: one model with only a linear term and one with a linear term and restricted cubic spline terms.

Models stratified by country, stage (metastatic, non-metastatic, unknown) and menopausal status at diagnosis and adjusted for age at diagnosis, attained level of education, physical activity, body mass index (modelled as restricted cubic spline), alcohol consumption reported at recruitment, smoking habit and intensity at recruitment, ever use of hormone for menopause at diagnosis, grade of differentiation, and tumor receptor status (ER, PR, HER2).

The values on the y-axis correspond to hazard ratios (HR) in a logarithmic (ln) scale. The values -0.4, -0.2, 0.0, 0.2, and 0.4 correspond, respectively, to the HRs 0.67, 0.82, 1.00, 1.22, and 1.49.
